# Macrophage-derived exosomal microRNA-501-3p promotes progression of pancreatic ductal adenocarcinoma through the TGFBR3-mediated TGF-β signaling pathway

**DOI:** 10.1186/s13046-019-1313-x

**Published:** 2019-07-15

**Authors:** Zi Yin, Tingting Ma, Bowen Huang, Lehang Lin, Yu Zhou, Jinhai Yan, Yiping Zou, Sheng Chen

**Affiliations:** 1 Department of General Surgery, Guangdong Provincial People’s Hospital, Guangdong Academy of Medical Sciences, No. 106, Zhongshan Er Road, Guangzhou, 510080 Guangdong Province People’s Republic of China; 2 Department of Obstetrics and Gynecology, Sun Yat-Sen Memorial Hospital of Sun Yat-Sen University, Guangzhou, 510120 People’s Republic of China; 3 Medical Research Center, Sun Yat-Sen Memorial Hospital of Sun Yat-Sen University, Guangzhou, 510120 People’s Republic of China; 4 Pathology Department, Guangdong Provincial People’s Hospital, Guangdong Academy of Medical Sciences, Guangzhou, 510080 People’s Republic of China

**Keywords:** Pancreatic ductal adenocarcinoma, Exosome, M2 macrophage, MicroRNA-501-3p, TGFBR3, TGF-β signaling pathway, Cell invasion, Metastasis, Angiogenesis

## Abstract

**Background:**

Exosomes from cancer cells or immune cells, carrying bio-macromolecules or microRNAs (miRNAs), participate in tumor pathogenesis and progression by modulating microenvironment. Our study aims to investigate the role of these microRNA-501-3p (miR-501-3p) containing exosomes derived from tumor-associated macrophage (TAM) in the progression of pancreatic ductal adenocarcinoma (PDAC).

**Methods:**

Firstly, the function of TAM recruitment in PDAC tissues was assessed, followed by identification of the effects of M2 macrophage-derived exosomes on PDAC cell activities and tumor formation and metastasis in mice. In silico analysis was conducted to predict differentially expressed genes and regulatory miRNAs related to PDAC treated with macrophages, which determined miR-501-3p and TGFBR3 for subsequent experiments. Next, gain- and loss-of-function experiments were performed to examine their role in PDAC progression with the involvement of the TGF-β signaling pathway.

**Results:**

TAM recruitment in PDAC tissues was associated with metastasis. Highly expressed miR-501-3p was observed in PDAC tissues and TAM-derived exosomes. Both M2 macrophage-derived exosomes and miR-501-3p promoted PDAC cell migration and invasion, as well as tumor formation and metastasis in nude mice. MiR-501-3p was verified to target TGFBR3. PDAC cells presented with down-regulated TGFBR3, which was further decreased in response to M2 macrophage treatment. TGF-β signaling pathway activation was implicated in the promotion of miR-501-3p in PDAC development. The suppression of macrophage-derived exosomal miR-501-3p resulted in the inhibition of tumor formation and metastasis in vivo.

**Conclusion:**

M2 macrophage-derived exosomal miR-501-3p inhibits tumor suppressor TGFBR3 gene and facilitates the development of PDAC by activating the TGF-β signaling pathway, which provides novel targets for the molecular treatment of PDAC.

**Electronic supplementary material:**

The online version of this article (10.1186/s13046-019-1313-x) contains supplementary material, which is available to authorized users.

## Background

Pancreatic cancer represents the seventh leading cause of death in relation to cancers, and the most common type is pancreatic ductal adenocarcinoma (PDAC), an infiltrating cancer with glandular differentiation originating from pancreatic ductal tree [1]. PDAC is a fatal malignancy with an 8% 5-year overall survival rate for cases of all stages; most of these cases have been confirmed as being stage IV at diagnosis and have a 3% 5-year overall survival [2]. Pancreaticoduodenectomy is the standard therapeutic method that is applied in PDAC patients, which might elongate the long-term survival [3]. However, the recurrence of PDAC is a common occurrence following resection surgery, which exacerbates and usually results in the development of liver metastasis, lung metastasis, peritoneal seeding, peripancreatic recurrence, and other distant metastases [4, 5]. Therefore, due to the propensity for resistance to systemic therapy and early metastasis, effective early detection and screening seem quite indispensable to prevent PDAC, which is currently inadequate [6]. Thus, there is an urgent need to identify promising biomarkers that regulate biological activities of PDAC cells, in order to develop new therapeutic modalities for PDAC with favorable and accurate prognosis.

Tumor-associated macrophages (TAMs), like M2 phenotype cells, are leukocytes that have a probability of infiltrating solid tumors, and stimulate cell invasion, proliferation and angiogenesis [7, 8]. Due to their contributory effect on lymphatic metastasis, M2-polarized TAMs have been proven to be associated with an unfavorable prognosis in pancreatic cancer [9]. Furthermore, exosomes exist in the circulation and tissue microenvironment [10] and it has been suggested that M2 macrophage-derived exosomes deliver a regulatory transfer of specific proteins or signaling to tumor cells to control their migration. An example of such includes the exosome-mediated transfer of ApoE protein from TAMs to the gastric cancer cells that results in the enhancement of cell migration [11]. Exosomes have been found to carry functional macromolecules and “exosomal shuttle RNAs” such as miRNA [12]. A previous study was highly suggestive of the feasibility of the use of plasma-based miRNA profiling as specific and sensitive biomarker assays for PDAC, which might enable the potential of clinic translation with effective improvements [13]. Previous studies have demonstrated the potential associations that exist between aberrantly expressed microRNAs (miRNAs) and target genes related to PDAC, which has also been linked to the development of miRNA-driven PDAC [14]. In terms of pancreatic cancer cells, microRNA-301a-3p (miR-301a-3p)-carrying exosomes were secreted under a hypoxic condition, whereby the macrophages were polarized to accelerate the cell migration, invasion, and epithelial-mesenchymal transition [15]. Similarly, there have been previously conducted studies on the promoting role of miR-501 in cervical cancer, and results found that its up-regulation augmented the cell proliferation, migration and invasion of cervical cancer through the regulation of the target gene of CYLD [16]. The loss of TGF-beta Receptor III (TGFBR3) expression primes clear-cell renal cell carcinoma cells with facilitated metastatic potential by diminishing TGF-β2 signaling, which was implicated in poor prognosis of patients [17]. TGFBR3 has been demonstrated to be regulated by several miRNAs, including miR-424 and miR-193–3p [18, 19]. Therefore, on the basis of the above findings, we hypothesized that TAM-derived exosomes played a role in the progression of PDAC, and that the mechanism of which may be involved in miR-501-3p and the TGFBR3-mediated TGF-β signaling pathway.

## Materials and methods

### Ethics statement

This study was conducted with the approval of the Ethical Committee of Guangdong Academy of Medical Sciences. Informed consent and required documentation were obtained from each patient and their respective guardians prior to the study. All animal procedures were conducted with strict accordance to the recommendations on the Guide for the Care and Use of Laboratory Animals of the National Institutes of Health.

### Study subjects

A total of 56 specimens were obtained from PDAC patients (38 males and 18 females, aged 32–75 years) who had received pancreatectomy in Guangdong Academy of Medical Sciences from June 2009 to September 2014. Prior to the pancreatectomy, the patients hadn’t received any chemotherapy or radiotherapy. The resected PDAC tissues were immediately preserved in the liquid nitrogen at − 80 °C. According to the criterion of World Health Organization (WHO) classification, the diagnosis of PDAC was independently verified by two pathologists. The above patients had their continuous follow-up for 3 years, with the follow-up deadline set for September 2017. The follow-up was performed by means of telephone interview, or outpatient visit. The overall survival (OS) was determined from the date of random grouping to the date of death due to any cause or the last follow-up visit.

### Cell lines

PDAC cell lines (PANC-1, BxPC-3, MIA Paca-2 and Capan-2), human microvascular endothelial cell (HMEC)-1 and THP-1 cells were purchased from American Type Culture Collection (ATCC, Manassas, VA, USA) (https://www.atcc.org/). The exosomes in the medium were removed through centrifugation at 100,000 g at 4 °C overnight [20]. PANC-1, BxPC-3, MIA Paca-2, Capan-2 and HMEC-1 cells were incubated in Dulbecco’s modified Eagle’s medium (DMEM, 31600–034, Hyclone, Logan, UT, USA) supplemented with 10% fetal bovine serum (FBS, 10099141, Gibco, Grand Island, NY, USA) [21, 22]. THP-1 cells were cultured in Roswell Park Memorial Institute (RPMI) 1640 medium [23] containing 10% FBS in a constant temperature incubator at 37 °C with 5% CO_2_ and sufficient humidity. Once the cell confluence reached 90%, the cells were incubated and passaged at a ratio of 1: 3–4. The cell lines used were all verified by short tandem repeat (STR) analysis and were free of mycoplasma contamination [15]. THP-1 cells were treated with 100 ng/mL phorbol 12-myristate 13-acetate (PMA, P8139, Sigma, St. Louis, Missouri, USA) for differentiation into macrophages for 24 h. Next, 100 ng/mL lipopolysaccharide (LPS, 8630, Sigma) and 20 ng/mL interferon-γ (IFN-γ, 285-IF, R&D, Minneapolis, MN, USA) were adopted to treat cells for 24 h, polarizing them into an M1 phenotype. Following treatment with 20 ng/mL interleukin-4 (IL-4, AF-200-04-5, Peprotech, Rocky Hill, NJ, USA) for 72 h, the cells were polarized into an M2 phenotype [24].

### Cell treatment and grouping

The procedures of cell transfection were described as follows. Human mononuclear macrophage line (THP-1) was regarded as a control group without any transfection and Mp-Exo as an experimental group with M2 macrophage exosomes. Once the cell confluence reached 80–90% in the PANC-1 and BxPC-3 cells, they were transfected according to the instructions provided on the Lipofectamine 2000 (11668–019, Invitrogen, New York, CA, USA). Subsequently, the cells were grouped according to Table 1 into negative control (NC) mimic group (transfection of miR-501-3p mimic NC), miR-501-3p mimic group (transfection of miR-501-3p mimic plasmids), Mp-Exo + NC inhibitor group (transfection of M2 macrophage-derived exosomes and miR-501-3p inhibitor NC), Mp-Exo + miR-501-3p inhibitor group (transfection of M2 macrophage-derived exosomes and miR-501-3p inhibitor), NC siRNA group (transfection of TGFBR3 siRNA NC), TGFBR3 siRNA group (transfection of TGFBR3 siRNA), NC inhibitor group (transfection of miR-501-3p inhibitor NC), miR-501-3p inhibitor group (transfection of miR-501-3p inhibitor), PBS + NC mimic group (transfection of M2 macrophage-derived exosome NC and miR-501-3p mimic NC) and Mp-Exo + NC mimic group (transfection of M2 macrophage-derived exosome and miR-501-3p mimic NC).

**Table 1:** Cell grouping information

	1		2		3		4		5		6				7			
	NC mimic	miR-501-3p mimic	Mp-Exo+NC inhibitor	Mp-Exo+miR-501-3p inhibitor	NC siRNA	TGFBR3 siRNA	NC inhibitor	miR-501-3p inhibitor	PBS+NC mimic	Mp-Exo+NC mimic	NC mimic+Vector	miR-501-3p mimic+Vector	NC mimic+OE-TGFBR3	miR-501-3p mimic+OE-TGFBR3	PBS+Vector	Mp-Exo+Vector	PBS+OE-TGFBR3	Mp-Exo+ OE-TGFBR3
PBS									±						±		±	
miR-501-3p mimic		±										±		±				
Mimic NC	±								±	±	±		±					
miR-501-3p inhibitor				±				±										
Inhibitor NC			±				±											
Mp-Exo			±	±						±						±		+
Vector											±	±			±	±		
NC siRNA					±													

Afterwards, the cells were assigned into NC mimic + vector group (transfection of miR-501-3p mimic NC and infection of TGFBR3 NC), miR-501-3p mimic + vector group (transfection of miR-501-3p mimic and infection of TGFBR3 NC), NC mimic + overexpression (oe)-TGFBR3 group (transfection of miR-501-3p mimic NC and infection of TGFBR3 adenovirus), miR-501-3p mimic + oe-TGFBR3 group (transfection of miR-501-3p mimic and infection of TGFBR3 adenovirus), PBS + vector group (treatment of M2 macrophage-derived exosome NC and infection of TGFBR3 adenovirus NC), Mp-Exo + vector group (treatment of M2 macrophage-derived exosome and infection of TGFBR3 adenovirus NC), PBS + oe-TGFBR3 group (treatment of M2 macrophage-derived exosome NC and infection of TGFBR3 adenovirus), and Mp-Exo + oe-TGFBR3 group (treatment of M2 macrophage-derived exosome and infection of TGFBR3 adenovirus). The miR-501-3p mimic, miR-501-3p inhibitor, and TGFBR3 siRNA (there were three siRNAs to be assessed) at a concentration of 50 nM and TGFBR3 overexpressing adenoviruses were all purchased from RiboBio Co., Ltd. (Guangzhou, China). The M2 macrophages were pretreated by 5 μM of exosome secretion inhibitor GW4869 (HY-19363, MCE, Monmouth Junction, NJ, USA). The experiment was conducted in triplicates.

### Isolation and characterization of exosomes

Once the M2 macrophage confluence reached 80–90%, the complete medium was discarded and replaced with fresh medium. Next, 30 mL of used cell culture medium was collected per cell line [20], out of which the exosomes were isolated through the method of differential centrifugation at 4 °C. After additional centrifugation at 4 °C at 100,000 g overnight, the exosomes were extracted in accordance with the instructions on the ExoQuick (System Bioscience, Mountain View, CA, USA). Next, the precipitation was washed with a large amount of PBS, followed by re-suspension in PBS, and was stored at − 80 °C for further use.

Subsequently, the exosomes were identified using transmission electron microscope. Firstly, 20 μL of exosomes were added dropwise on a copper mesh and allowed to stand for 3 min. The liquid was then blotted from the side with filter paper. Next, 30 μL of phosphotungstic acid solution (pH 6.8) was added dropwise to counterstain exosomes for 5 min at room temperature. After being baked by an incandescent lamp, the exosomes were photographed under a transmission electron microscope. Particle size analysis [25] was performed using nanoparticle tracking analysis (NS300, Malvern Instruments Ltd., Malvern, UK). Western blot analysis was adopted to identify exosome surface markers. The exosomal suspension was concentrated and the protein content was determined using the bicinchoninic acid (BCA) kit (23227, Thermo Fisher Scientific, Waltham, MA, USA). Following denaturation, sodium dodecyl sulfate-polyacrylamide gel electrophoresis (SDS-PAGE) was carried out for protein separation. Afterwards, the samples were transferred onto membranes, and the expression of specific marker proteins of exosomes was examined such as tumor susceptibility gene 101 (TSG101), CD63 and CD81 [25].

### Cell counting kit-8 (CCK-8)

The experimental procedures were carried out according to the instructions provided on the CCK-8 kit (C0037, Beyotime Institute of Biotechnology, Shanghai, China). The cells were seeded at a density of 2 × 10^3^ cells/well into 96-well plates and incubated for 24 h. Subsequently, 10 μL of CCK-8 reagent was added to 100 μL of complete medium for further incubation. The absorbance was measured at 450 nm by a Multiskan FC plate reader (51119100, Thermo Fisher Scientific) at different time points (0 h, 24 h, 36 h, 48 h and 72 h). The experiment was repeated three times with triplicates.

### Transwell assays

Cell migration experiments were conducted as follows. Firstly, the cells were seeded at a cell density of 5 × 10^4^ cells/mL into the Transwell chamber (pore size of filter = 8 μm) with 200 μL per chamber. The basolateral chamber was then seeded with corresponding cells or 500 μL of 10% FBS medium. The experiment was repeated three times with triplicates. Following incubation in a 37 °C incubator with 5% CO_2_ for 24 h, the Transwell chamber was removed and fixed with 4% paraformaldehyde-PBS solution at 4 °C. Next, crystal violet staining solution (C0121, Beyotime Institute of Biotechnology) was added for 20-min staining. Once the cells on the surface inside the chamber had been wiped off, the cells were observed under an inverted fluorescence microscope (TE2000, Nikon, China). Subsequently, 5 visual fields were randomly selected and photographed and the average number of cells penetrating through the chamber was calculated. The experiment was conducted in triplicates.

The above procedure was followed by cell invasion experiments. Briefly, the stock solution of Matrigel (40111ES08, Yeasen Company, Shanghai, China) was diluted into 1/3 by DMEM. The solution was then used to coat the apical chamber of the Transwell (3413, Unique Biotechnology Co. Ltd., Beijing, China), followed by incubation in a 37 °C incubator for 4–5 h until Matrigel coagulation was obtained. The cell suspension of transfected cells was diluted with 100 μL of serum-free medium to a density of about 1 × 10^6^ cells/mL. Following inoculation, 500 μL of DMEM containing 10% FBS was added to the basolateral chamber. The experiment was repeated three times with triplicates. After incubation at 37 °C and 5% CO_2_ for 24 h, the Transwell chamber was fixed in 4% paraformaldehyde-PBS solution and stained with crystal violet for 5 min. The cells on the inner surface were wiped off. Next, 5 fields were randomly selected and photographed under an inverted fluorescence microscope (TE2000, Nikon, China), after which the average number of cells penetrating through the chamber was calculated. The experiment was conducted in triplicates.

### Matrigel tube formation assay

Matrigel (354234, Shanghai Shanran Biotechnology Co., Ltd., Shanghai, China) was placed in a freezer at 4 °C overnight, after which it was melted into a yellow gelatinous liquid. Next, 10 μL (thickness = 0.5 mm) of yellow gelatinous liquid was aspirated with a pre-cooled micropipette and quickly added onto the pre-cooled angiogenic slides (81506, ibidi, Martinsried, Germany). The slides were then incubated in a humidity chamber in a 37 °C incubator for about 30 min and allowed to solidify. After an incubation period of 24 h, the cells were collected and starved in serum-free for 1 h, followed by re-suspension in DMEM to prepare a cell suspension. Subsequently, 50 μL of the cell suspension with a density of 2 × 10^5^ cells/mL was seeded onto the Matrigel-coated slides. All investigations involved at least 3 wells, each repeated in triplicate. After incubation for 12 h, the slides were photographed under a Leica inverted phase contrast microscope. The number of intact capillary lumens constituted by cells was calculated by Image-Pro Plus software (version 6.0) under a microscope (100 ×). At least 3 fields were calculated in each group. The experiment was repeated in triplicates.

### Western blot analysis

Total protein of tissue, cell or exosome was extracted using radio-immunoprecipitation assay (RIPA) lysis buffer (R0010, Solarbio, Shanghai, China). The supernatant was collected following centrifugation (12,000 rpm) at 4 °C for 15 min, after which the protein concentration was determined and quantified using a BCA kit (20201 ES76, Yeasen Company, Shanghai, China). The protein was separated with the use of acrylamide gel electrophoresis and transferred onto a polyvinylidene fluoride (PVDF) membrane. After blockade by 5% skim milk, blots were probed with diluted primary antibodies: rabbit anti-TSG101 (ab30871, 1: 1000), CD63 (ab68418, 1: 1000), CD81 (ab109201, 1: 1000), vascular endothelial growth factor A (VEGFA) (ab46154, 1: 1000), vascular endothelial growth factor receptor 2 (VEGFR-2) (ab11939, 1: 1000), angiopoietin 2 (ANG2) (ab8452, 1: 500), phosphatidylinositol glycan anchor biosynthesis, class F (PIGF) (ab74778, 1: 1000), intercellular adhesion molecule 1 (ICAM1) (ab53013/179707, 1: 2000/1: 1000), cleaved caspase 3 (ab49822, 1: 500), cleaved-PARP1 (ab32064, 1: 1000), Musashi RNA binding protein 2 (MSI2) (ab76148, 1: 1000), Fibronectin (ab2413, 1: 1000), TGFBR3 (ab97459, 1: 1000), TGF-beta Receptor I (TGFBR1) (ab31013, 1: 1000), TGF-beta Receptor II (TGFBR2) (ab186838, 1: 1000), t-SMAD3 (ab40854, 1: 2000), p-SMAD3 (ab52903, 1: 2000), mouse anti-E-Cadherin (ab76055,1: 1000) and glyceraldehyde-3-phosphate dehydrogenase (GAPDH) (ab8245, 1: 5000) at 4 °C overnight. Subsequently, the membrane was incubated with horseradish peroxidase (HRP)-labeled goat anti-rabbit IgG (ab205718, 1: 20000) or goat anti-mouse (ab6789, 1: 5000) dilution for 1 h at room temperature. The aforementioned antibodies were all purchased from Abcam Inc. (Cambridge, UK). Quantitative analysis of protein was conducted using Image J 1.48u software (National Institutes of Health, Bethesda, MA, USA). The protein level was expressed as the ratio of gray values of target bands to that of internal reference GAPDH.

### Reverse transcription quantitative polymerase chain reaction (RT-qPCR)

The total RNA of cells and tissues in addition to the exosome RNA was extracted using the RNA extraction kit (AM1552, Thermo Fisher Scientific) according to the instructions of RNA extraction kit, after which the RNA concentrations were determined. The primers used in this study were synthesized by Takara (Dalian, Liaoning, China) (Table 2). The relative transcriptional level of the target gene was calculated using the 2^-△△CT^ method [26] with GAPDH and U6 used as the internal references: △△Ct = △Ct _experimental group_ - △Ct _control group_, △Ct = Ct _target gene_ - Ct _internal reference_.

**Table 2:** Primer sequences for RT-qPCR

Gene	Sequence (5'-3')	
CD206	F: CAAGGAAGGTTGGCATTTGT	R: CCTTTCAGTCCTTTGCAAGC
CD68	F: GCTACATGGCGGTGGAGTACAA	R: ATGATGAGAGGCAGCAAGATGG
iNOS	F: ACAGGAGGGGTTAAAGCTGC	R:TTGTCTCCAAGGGACCAGG
Arginase	F: TTGGCAATTGGAAGCATCTCTGGC	R: TCCACTTGTGGTTGTCAGTGGAGT
GAPDH	F: ATGGAGAAGGCTGGGGCTC	R: AAGTTGTCATGGATGACCTTG
TGFBR3	F: CTGAAATCGTGGTGTTTAATTG	R: GCTCC ATGTTGAAGGTGATG
CD133	F: ACCAGGTAAGAACCCGGATCAA	R: CAAGAATTCCGCCTCCTAGCACT
NANOG	F: TGCCTCACACGGAGACTGTC	R: TGCTATTCTTCGGCCAGTTG
OCT4	F: CTGAAGCAGAAGAGGATCAC	R: GACCACATCCTTCTCGAGCC
Cyclin A2	F: TTATTGCTGGAGCTGCCTTT	R: ACTGTTGTGCATGCTGTGGT
Cyclin D1	F: ACCTGGATGCTGGAGGTCT	R: GCTCCATTTGCAGCAGCTC
Cyclin D2	F: TTTGCCATGTACCCACCGTC	R: AGGGCATCACAAGTGAGCG
Cyclin E1	F: GAAGAGGAAGGCAAACGTGA	R:TGCACGTTGAGTTTGGGTAA
p16	F: CATAGATGCCGCGGAAGGT	R:GGATTAGGGCTTCCTCTTGGA
p19	F: GTC CCA GCC AGC CAT GGCAG	R: GGC CTT GCT GGG CCA TGG AG
p21	F: AGTCAGTTCCTTGTGGAGCC	R: CATGGGTTCTGACGGACAT
p53	F: GAGCCCCCTCTGAGTCAG	R: GCAAAACATCTTGTTGAG
miR-501-3p	F: GCGGCGGAATGCACCCGGGCAAG	R: GTGCAGGGTCCGAGGT
U6	F: GCTTCGGCAGCACATATACTAAAAT	R: CGCTTCACGAATTTGCGTGTCAT

Note: *RT-qPCR* Reverse transcription quantitative polymerase chain reaction, *F* Forward, *R* Reverse.

### Bioinformatics prediction

The GEO database (https://www.ncbi.nlm.nih.gov/geo/) was used to retrieve the gene expression data related to PDAC, after which differential analysis was conducted using limma package of R language. Next, TGFBR3 expression in the TCGA database was detected via GEPIA database (http://gepia.cancer-pku.cn/index.html). Subsequently, the potential miRNAs regulating TGFBR3 were predicted using the TargetScan database (http://www.targetscan.org/vert_71/).

### Dual luciferase reporter gene assay

The targeting relationship of TGFBR3 and miR-501-3p was predicted using an online website, after which it was further confirmed by dual luciferase reporter gene assay. The dual luciferase reporter vector of TGFBR3 and the mutants of binding sites of TGFBR3 to the miR-501-3p were designed separately: pGL3-TGFBR3-wild type (Wt) and pGL3-TGFBR3-mutation (Mut). The two reporter plasmids were co-transfected into HEK293 cells with the plasmid that had overexpressed miR-501-3p and pRL-TK (internal reference plasmid expressing Renilla luciferase). After a 24 h transfection, a dual luciferase reporter system (Dual-Luciferase® Reporter Assay System, E1910, Promega, Madison, WI, USA) was adopted to determine luciferase activity, which was represented by the ratio of Firefly luciferase to Renilla luciferase. The experiment was conducted in triplicates.

### Tumorigenicity in nude mice

A total of 56 male BALB/c nude mice (aged 3–6 weeks and weighing 16–22 g), purchased from Guangdong Medical Laboratory Animal Center (Foshan, Guangdong, China), were housed in laminar flow cabinets under specific pathogen-free (SPF) conditions, subjected to regular indoor UV irradiation. The mice were kept under controlled environmental conditions in a disinfected cage, with disinfected padding, drinking water and food, at room temperature of 24–26 °C and relative humidity of 40–60%. The nude mice were then divided into PANC-1 + saline group (PANC-1 cells treated with saline), PANC-1 + Mp-Exo group (PANC-1 cells treated with M2 macrophage-derived exosome), BxPC-3 + PBS group (BxPC-3 cells treated with PBS), and BxPC-3 + Mp-Exo group (BxPC-3 cells treated with M2 macrophage-derived exosome). There were 7 mice in each group. PANC-1 and BxPC-3 cells in logarithmic growth phase were collected and resuspended at a density of 1 × 10^6^ cells/100 μL with PBS. Subsequently, 100 μL of the cell suspension was subcutaneously inoculated into the right groin of nude mice, and Mp-Exo or normal saline was injected into the tail caudal vein. After 4 weeks of inoculation, the tumor tissue was dissected. The tumor tissue of the PANC-1 + saline group was transplanted into the pancreas capsule of nude mice, followed by intravenous injection of Mp-Exo or normal saline into caudal vein. After 6 weeks, the size of the dissected tumor was measured according to the following formula: tumor volume = (length × width × height) × 0.5. The tumor weight was weighed with a balance. The liver and lung tissues were isolated and the metastatic nodules were counted. The tumor tissues were then fixed in 10% formaldehyde, routinely dehydrated, embedded in paraffin, and sliced into 4-μm-thick sections. Afterwards, the remaining mice were assigned into PANC-1 + Exo-NC antagomiR group, PANC-1 + Exo-miR-501-3p antagomiR group, BxPC-3 + Exo-NC antagomiR group and BxPC-3 + Exo-miR-501-3p antagomiR group, with 7 mice in each group. The exosomes were extracted following the transfection of M2 macrophages with NC antagomiR or miR-501-3p antagomiR. The tumor formation experiments that were conducted subsequently followed the same procedure as the ones mentioned above.

### Hematoxylin-eosin (HE) staining

The paraffin-embedded sections that had been prepared were dewaxed, after which hematoxylin was used to stain the nucleus for 5 min. After dissimilation with the use of 1% ethanol-hydrochloric acid for 30 s, the sections were observed under a microscope. The cytoplasm was stained with eosin. Finally, the sections were sealed with neutral gum and photographed under an optical microscope.

### Immunohistochemistry

Frozen sections were rewarmed at room temperature, and fixed in ice-cold acetone. The antigen retrieved sections were treated with 0.3% tritonX-100 and blocked with 5% BSA at room temperature. The sections were then incubated with diluted primary antibody F4/80 (Ab100790, 1: 100, Abcam Inc., Cambridge, UK) at 4 °C overnight. The following day, the rewarmed sections were incubated with HRP labeled goat anti-rabbit IgG antibody (ab6721, 1: 1000, Abcam Inc., Cambridge, UK) at room temperature for 30 min. Color reaction was developed using diaminobenzidine chromogen (DAB) solution for 3 min. After counterstaining of hematoxylin for nucleus, the sections were subjected to dehydration, permeabilization and mounting. Brown-yellow particles represented positive expression of the target protein. Finally, five randomly fields were selected from each section and observed under a microscope.

### Statistical analysis

SPSS 21.0 statistical software (IBM Corp. Armonk, NY, USA) was used for data processing. The measurement data were expressed as mean ± standard deviation. The comparison between two groups was performed using independent sample *t* test. One-way analysis of variance (ANOVA) was employed for comparison among multiple groups, followed by Tukey’s post-hoc test for pairwise comparison. The data at different time points were compared using repeated measures ANOVA. Kaplan-Meier method and the log-rank test were employed to analyze the relationship between TAM recruitment in PDAC and overall survival of patients. *p <* 0.05 was considered to be statistically significant.

## Results

### Tumor-associated macrophage recruitment in PDAC is associated with cancer metastasis

**Fig. 1:**
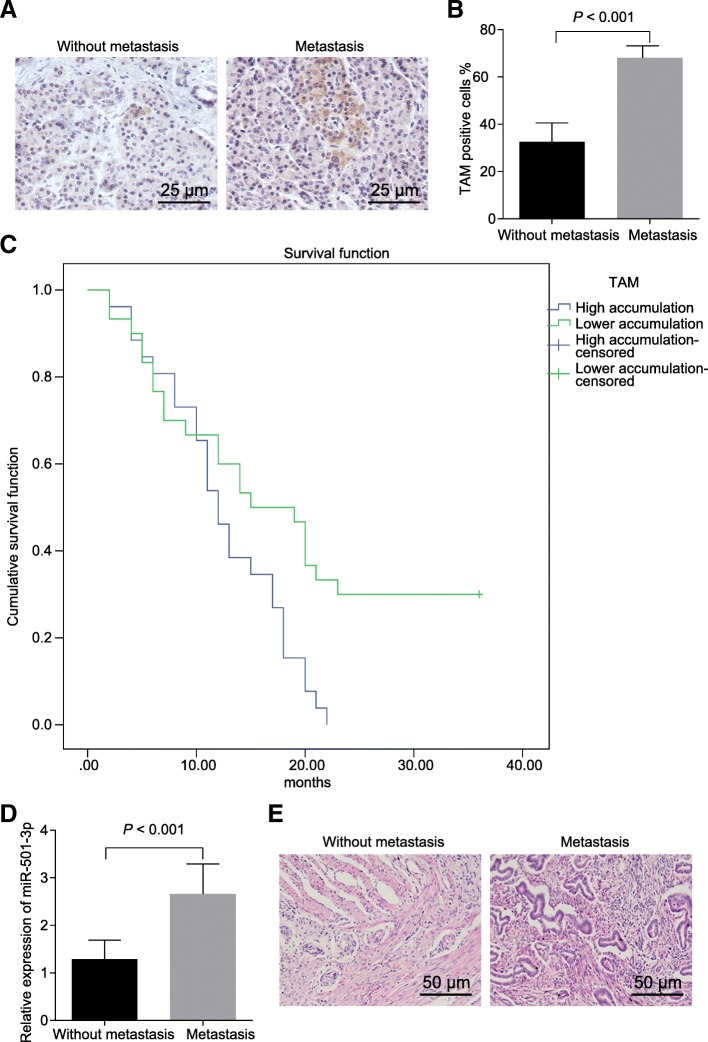
Tumor-associated macrophage recruitment in PDAC associates with cancer metastasis. **a** and **b** immunohistochemical staining and quantitative analysis of F4/80 positive expression in metastatic and non-metastatic PDAC tissues. **c** correlation of TAM recruitment with survival curve of PDAC patients. **d** relative expression of miR-501-3p in metastatic and non-metastatic PDAC patients. **e** HE staining for PDAC tissues. * *p <* 0.05. The measurement data were expressed as mean ± standard deviation. Data between two groups were analyzed by independent sample *t* test. Kaplan-Meier method and the log-rank test were employed to analyze the relationship between TAM recruitment in PDAC and overall survival

There were 42 cases of metastatic tissues, and 12 cases of non-metastatic tissues among subjects. Immunohistochemical results (Fig. 1a & b) showed that TAM (F4/80 positive) recruitment was significantly increased in PDAC tissue of metastatic patients compared with non-metastatic patients. According to the mean value of TAM, the patients were classified into a group with high enriched level of TAM and the other group with low level. Survival analysis (Fig. 1c) revealed that patients with highly recruited TAM had a lower cumulative survival rate. The tissue samples obtained from the metastatic patients presented with higher expression of miR-501-3p (Fig. 1d). The HE staining analysis for PDAC tissues is displayed in Fig. 1e. It has been reported that miR-501-3p in M2 macrophages (F4/80 positive) was higher than that in M1 macrophages [24].

### M2 macrophage-derived exosomes promote the migration and invasion of PDAC cells

**Fig. 2:**
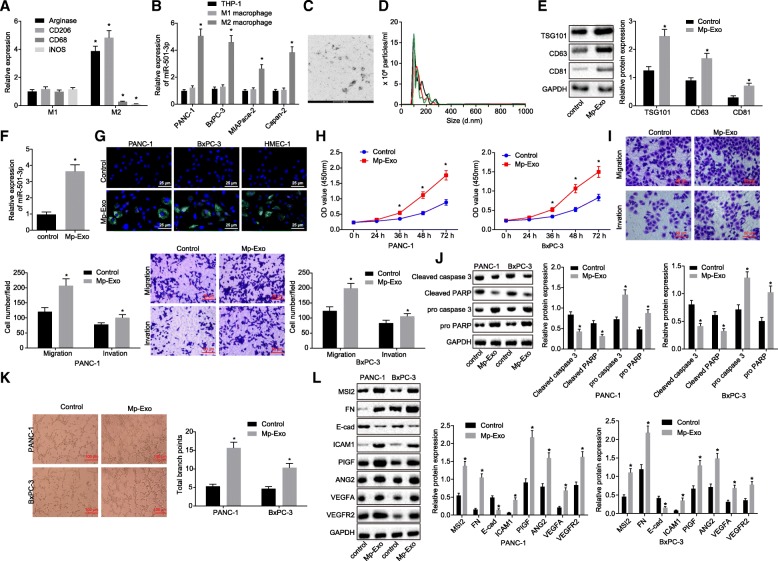
M2 macrophage-derived exosomes promote migration and invasion of PDAC cells. **a** the expression of marker genes for M1 and M2 macrophages determined by RT-qPCR. **b** the expression of miR-501-3p in PDAC cells determined by RT-qPCR. **c** identification of exosome structure under a transmission electron microscope (Scale bar = 100 nm). **d** detection of the size and number of exosomes (3 colors represent 3 replicates) by Nanoparticle tracking analysis. **e** Western blot analysis of TSG101, CD63 and CD81 protein after Mp-Exo treatment; evaluation of the expression of M2 macrophage exosomal markers when compared with that in the control group without any transfection. **f** miR-501-3p expression in response to Mp-Exo treatment determined by RT-qPCR. **g** fluorescence staining analysis of PKH-67 labeled Mp-Exo exosome internalization in PANC-1, BxPC-3 and HMEC-1 cells (Scale bar = 25 μM). **h** proliferation of PANC-1 and BxPC-3 cells after Mp-Exo treatment was examined using CCK-8 assay. **i** migration and invasive ability of PANC-1 and BxPC-3 cells after Mp-Exo treatment was examined by Transwell assay (Scale bar = 50 μM). **j** Western blot analysis of pro/cleaved caspase 3 and pro/cleaved PARP protein in PANC-1 and BxPC-3 cells after Mp-Exo treatment. **k**. In vitro tube formation assay was used to examine the HMEC-1 tube formation ability and quantitative analysis of branch points in PANC-1 and BxPC-3 cells (Scale bar = 100 μM). **l**. Western blot analysis was applied to detect migration, invasion and angiogenesis-related proteins in PANC-1 and BxPC-3 cells after Mp-Exo treatment. * *p <* 0.05 vs. M1 macrophage, THP-1 cells or control (cells without Mp-Exo treatment). The measurement data were expressed as mean ± standard deviation. Data between two groups were analyzed by independent sample *t* test. One-way ANOVA was employed for comparison among multiple groups, followed by Tukey’s post-hoc test. The data at different time points were compared using repeated measures ANOVA, followed by Tukey’s post-hoc test. Cell experiments were repeated three times

It has been reported that macrophages can regulate the development of PDAC through miRNAs carried by exosomes [24]. We evaluated whether the effect of M2 macrophages on PDAC cells was through exosomes to further understand the effects of M2 macrophages on PDAC. THP-1 cells were converted to M1 phenotype macrophages following treatment with LPS and γ-IFN, expressing CD68 and iNOS (M1 macrophage phenotype marker genes). After treatment with IL-4, they were converted to M2 phenotype macrophages expressing Arginase and CD206 (M2 macrophage phenotype marker genes) (Fig. 2a) (*p <* 0.05). The expression of miR-501-3p was measured following co-culture of M2 macrophages with PDAC cell lines. The results showed that relative to the THP-1 cells co-cultured with PDAC cells, there was a significant up-regulation in the expression of miR-501-3p in the co-culture of M2 macrophages and PDAC cell lines (Fig. 2b) (*p <* 0.05). PANC-1 and BxPC-3 PDAC cell lines with the highest expression of miR-501-3p were selected for subsequent experiments.

Subsequently, the purified THP-1 exosomes were extracted as the control group and purified M2 macrophage exosomes were extracted as the experiment group. After observation under a transmission electron microscope, it was found that the supernatant of M2 macrophage cell culture contained exosomes. The shape of exosomes was solid and dense, with typical two-layer membrane structure, presenting a disc or cup shape with an average diameter of 90 nm (Fig. 2c & d). Once the marker genes of M2 macrophage exosomes were detected, we found that the extracted exosomes were M2 macrophage exosomes (Additional file 2: Figure S2). In addition, the protein levels of exosomal markers TSG101, CD63 and CD81 were determined by Western blot analysis, the results of which revealed a significant increase in the protein levels of TSG101, CD63 and CD81 following Mp-Exo treatment, which further confirmed the successful extraction of exosomes (Fig. 2e). The expression of miR-501-3p in Mp-Exo was significantly increased in comparison to that in the THP-1 cells-derived exosomes (Fig. 2f). PKH67-labeled Mp-Exo was co-cultured with PANC-1 and BxPC-3 cells for 48 h, and the endocytosis of exosomes in PANC-1 and BxPC-3 and HMEC-1 cells was observed under an inverted fluorescence microscope. PKH67-labeled exosomes presenting green fluorescence could be observed in PANC-1 and BxPC-3 cells co-cultured with exosomes, while the cells without culturing of exosomes presented with DAPI-induced blue fluorescence (Fig. 2g). After 48 h of co-culture of PANC-1 and BxPC-3 cells with Mp-Exo, and CCK-8, Transwell assay and Western blot analysis were employed for the purpose of examining cell proliferation, migration and invasion and apoptosis. Compared with the exosomes from THP-1, PANC-1 and BxPC-3 cells with Mp-Exo treatment presented with accelerated proliferation of PDAC cells at 48 h and 72 h (Fig. 2h) (*p <* 0.01), and the number of migrating cells and invasive PDAC cells was significantly increased (Fig. 2i) (*p <* 0.01). Moreover, levels of apoptosis-related proteins (cleaved caspase 3 and cleaved PARP) were significantly decreased in PANC-1 and BxPC-3 cells that had received treatment with Mp-Exo, suggesting the inhibition of apoptosis by Mp-Exo (Fig. 2j) (*p <* 0.05).

In addition, the tube formation assay data revealed that HMEC-1 cells co-cultured with Mp-Exo presented with facilitated tubule formation (Fig. 2k) (*p <* 0.05). Western blot analysis was conducted again to determine the levels of migration-related proteins (ICAM-1, MSI2, E-cadherin, Fibronectin) and angiogenesis-related proteins (VEGFA, VEGFR2, ANG2, PIGF), which were all found to be elevated in Mp-Exo treated cells; however, the expression of E-cadherin was down-regulated in Mp-Exo treated cells (Fig. 2l) (*p <* 0.05).

The aforementioned results suggested that Mp-Exo can promote the proliferation, migration and invasion of PANC-1 and BxPC-3 cells, while enhancing the tube formation ability of HMEC-1 cells, and inhibiting the apoptosis.

### M2 macrophage-derived exosomes promote tumor formation and metastasis in nude mice

**Fig. 3:**
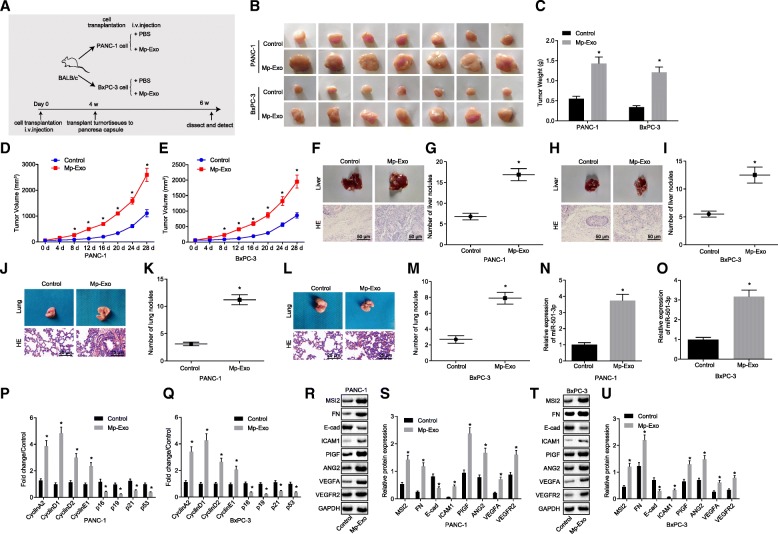
M2 macrophage-derived exosomes promote tumor formation and metastasis in nude mice. **a** a sketch map of tumor formation in nude mice. **b-e** xenograft tumors and quantitative analysis of tumor mass after Mp-Exo treatment (*n* = 7). **f-m** HE staining analysis and quantitative analysis of liver and lung nodules after Mp-Exo treatment (n = 7). **n-o** the expression of miR-501-3p in subcutaneous tumors of nude mice determined by RT-qPCR (n = 7). **p** and **q** cell cycle-related gene expression in subcutaneous tumors of nude mice was determined by RT-qPCR. **r**-**u** Western blot analysis was conducted to detect migration, invasion and angiogenesis-related proteins in response to Mp-Exo treatment. * *p <* 0.05 vs. control (cells without Mp-Exo treatment). The measurement data were expressed as mean ± standard deviation. Data between two groups were analyzed by *t* test. The data at different time points were compared using repeated measures ANOVA, followed by Tukey’s post-hoc test

Two PDAC cell lines (PANC-1 and BxPC-3) were injected into nude mice to establish an in vivo PDAC mouse model. Compared with the nude mice without Mp-Exo treatment, there was an evident increase in the weight and volume of subcutaneous tumors in nude mice following Mp-Exo treatment (Fig. 3a-e) (*p <* 0.05). Moreover, there was an increase in the number of metastatic liver and lung nodules (Fig. 3f-m) (*p <* 0.05). Meanwhile, the miR-501-3p expression was up-regulated in subcutaneous tumor tissues (Fig. 3n & o) (*p <* 0.01). The mRNA expression of cell cycle-related genes cyclinA2, D1, D2, E1 was significantly elevated, and that of the cell cycle inhibition-related genes p19 and p21 was reduced (Fig. 3p & q) (*p <* 0.05). The expression of migration-related proteins and angiogenesis-related proteins was determined, the results of which showed that the expression of ICAM-1, MSI2, Fibronectin, VEGFA, VEGFR2, ANG2 and PIGF was up-regulated, and E-cadherin was down-regulated, when compared to control group (Fig. 3r-u) (*p <* 0.05). These aforementioned results suggest that Mp-Exo can enhance the tumorigenic ability of PANC-1 and BxPC-3 cells, in addition to metastatic ability of tumor cells in vivo.

### Exosomal miR-501-3p promotes migration, invasion and tube formation of PDAC cells

**Fig. 4:**
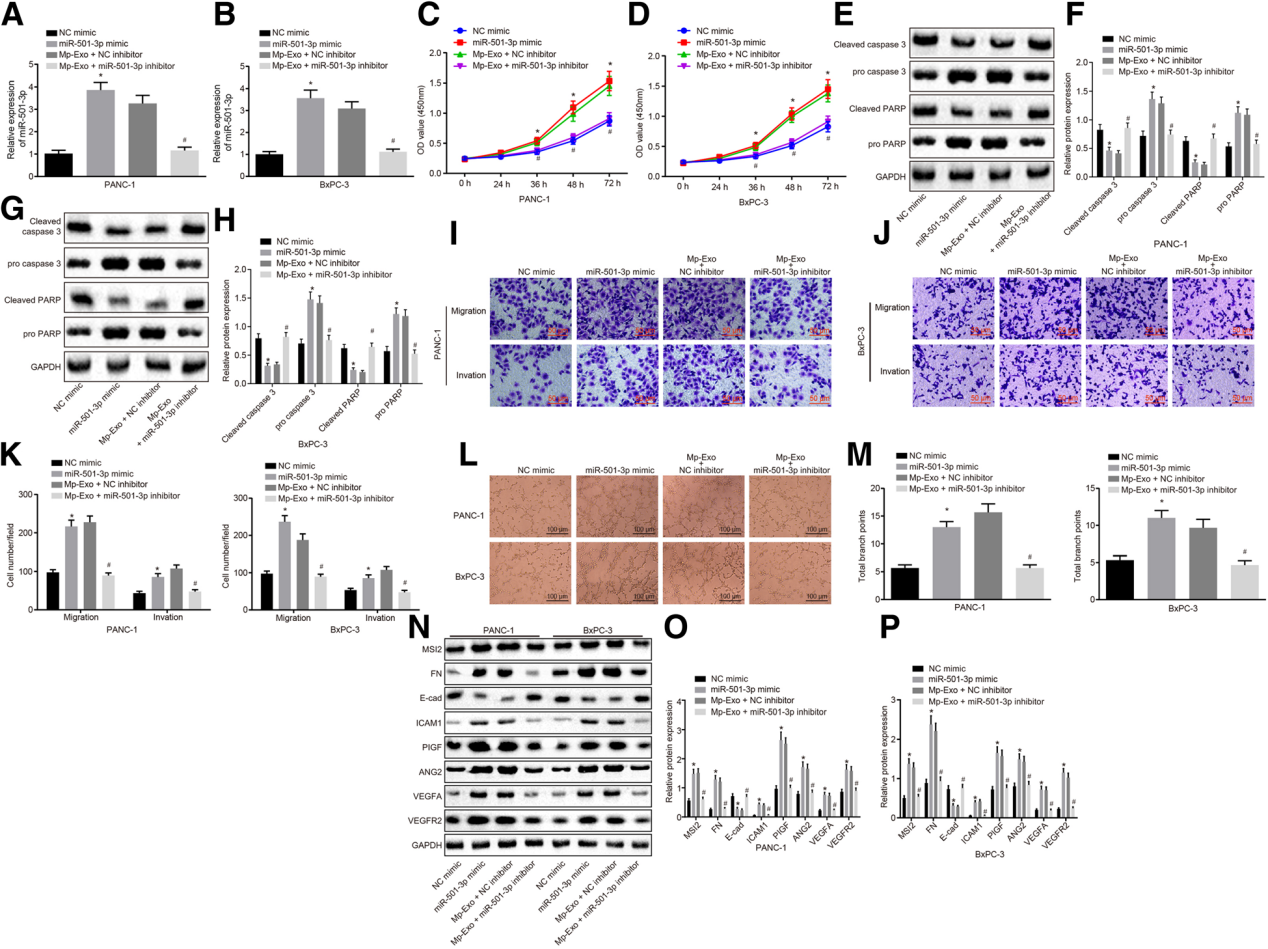
MiR-501-3p from M2 macrophage-derived exosomes promotes migration and invasion of PDAC cells. **a** and **b** expression of miR-501-3p in PANC-1 and BxPC-3 cells treated with miR-501-3p mimic and Mp-Exo + miR-501-3p inhibitor was determined by RT-qPCR. **c** and **d** proliferation of PANC-1 and BxPC-3 cells treated with miR-501-3p mimic and Mp-Exo + miR-501-3p inhibitor was examined using CCK-8 assay. **e-h** Western blot analysis of cleaved caspase 3 and cleaved PARP proteins in PANC-1 and BxPC-3 cells treated with miR-501-3p mimic and Mp-Exo + miR-501-3p inhibitor. **i-k** migration and invasion of PANC-1 and BxPC-3 cells treated with miR-501-3p mimic and Mp-Exo + miR-501-3p inhibitor were examined by Transwell assay (Scale bar = 50 μM). **l** and **m** In vitro tube formation assay was applied to assess the HMEC-1 tube formation ability and quantitative analysis of branch points in PANC-1 and BxPC-3 cells treated with miR-501-3p mimic and Mp-Exo + miR-501-3p inhibitor (Scale bar = 100 μM). **n-p** Western blot analysis was applied to detect migration, invasion and angiogenesis-related proteins in response to miR-501-3p mimic and Mp-Exo + miR-501-3p inhibitor. * *p <* 0.05 vs. the NC mimic group. # *p <* 0.05 vs. the Mp-Exo + NC mimic group. The measurement data were expressed as mean ± standard deviation. One-way ANOVA was employed for comparison among multiple groups, followed by Tukey’s post-hoc test. The data at different time points were compared using repeated measures ANOVA, followed by Tukey’s post-hoc test. Cell experiments were repeated three times

The aforementioned results revealed that miR-501-3p was differentially expressed when exosomes acted on PDAC cells. Therefore, we speculated that miR-501-3p might have an effect on PDAC cells. The expression of miR-501-3p following transfection in PANC-1 and BxPC-3 cells is illustrated in Fig. 4a & b. Following transfection with miR-501-3p mimic in PAR-1 and BxPC-3 cells, the results from CCK-8, Transwell assay and Western blot analysis revealed that miR-501-3p overexpression elevated the expression of miR-501-3p (Additional file 1: Figure S1A) and promoted the proliferation of PANC-1 and BxPC-3 cells (Fig. 4c & d) (*p <* 0.05), as well as migration and invasion ability (Fig. 4i-k) (*p <* 0.05), while inhibiting their apoptosis (Fig. 4e-h) (*p <* 0.05). In addition, the protein levels of ICAM-1, MSI2, Fibronectin, VEGFA, VEGFR2, ANG2, and PIGF were significantly up-regulated, and those of E-cadherin were down-regulated (Fig. 4n-p) (*p <* 0.05). MiR-501-3p mimic presented with evident promotion in HMEC-1 cell tube formation ability (Fig. 4l & m) (*p <* 0.05). These results in the PDAC cells with inhibited miR-501-3p expression in Mp-Exo were opposite to those induced by miR-501-3p mimic and Mp-Exo, indicating that miR-501-3p and Mp-Exo had a consistent effect on PDAC cells (PANC-1 and BxPC-3) and HMEC-1 cells. The effect of Mp-Exo on PDAC cells (PANC-1, BxPC-3) and HMEC-1 cells can be blocked following the inhibition of miR-501-3p expression in Mp-Exo.

### In silico analysis for differentially expressed genes and regulatory miRNAs related to PDAC treated with macrophages

**Fig. 5:**
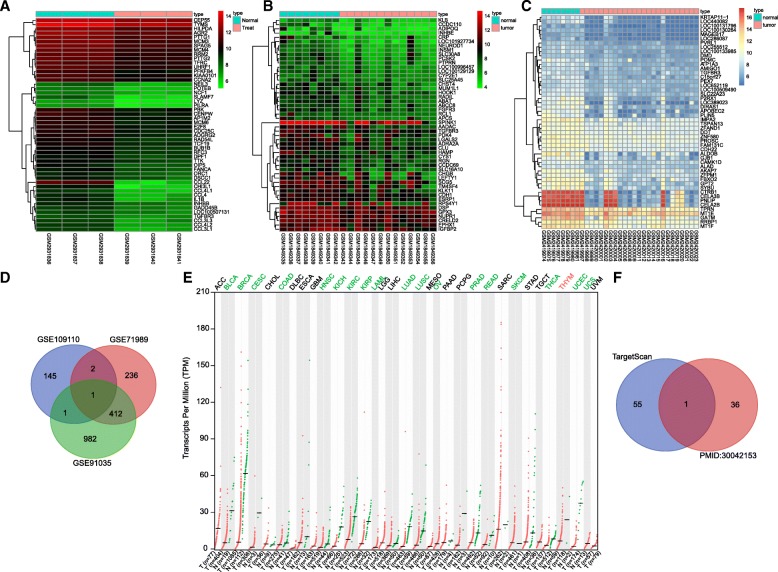
In silico analysis for differentially expressed genes and regulatory miRNAs related to PDAC treated with macrophages. **a-c** heat maps of differentially expressed genes obtained from gene expression datasets with macrophage-treated PDAC samples and common PDAC datasets. The abscissa represents the sample number, and the ordinate represents the differential expressed gene; the histogram at the left refers to gene expression cluster, and the histogram at the upper right refers to color gradation. **d** the intersection of the gene expression datasets analysis. The three circles in the panel represent the down-regulated genes in the three gene expression datasets, and the middle part represents the intersection of the three sets of data. **e** the expression of TGFBR3 in each tumor data of TCGA database. The abscissa indicates the sample type and tumor type, and the ordinate indicates the gene expression. **f** prediction of regulatory miRNAs of TGFBR3. The two sets of data in the panel are retrieved from the TargetScan database prediction and the study revealing miRNAs expressed in macrophage exosomes, and the middle part represents the intersection of the two sets of data

To identify the target gene of miR-501-3p, we retrieved a sequencing gene expression datasets GSE109110 with treatment of PDAC with macrophages in the GEO database [27]. The chip included PDAC samples before and after the treatment with macrophages, during which time the gene transcript difference between the two samples in the chip was performed. The gene expression dataset revealed that 249 genes were differentially expressed in PDAC samples treated with macrophages (Table 3). Among these differentially expressed genes, we noticed that there were 149 down-regulated genes in PDAC samples treated with macrophages. As illustrated in Fig. 5a, a heat map of 50 significantly down-regulated genes was obtained. In addition, in the GEO database, we obtained data from two PDAC sequencing datasets, both of which included normal pancreatic samples and PDAC samples. Subsequently, 50 differentially expressed genes were identified, whose expression levels were significantly down-regulated in the PDAC samples compared to those of the normal control samples (Fig. 5b & c). Furthermore, the down-regulated genes in the three chips were intersected (Fig. 5d), and it was found that, among the three sets of data, only one gene, TGFBR3, was present in the three sets. The expression level of TGFBR3 in TCGA database was further analyzed (Fig. 5e) and the results revealed that there was a poor expression in the TGFBR3 gene in almost all tumor samples. These analyses indicated that in addition to there being a low expression of TGFBR3 in PDAC, it was further decreased in response to macrophage treatment. It is suggested that the effect of macrophages on PDAC is highly likely to be achieved by regulating the TGFBR3 gene. The TargetScan database was used to predict the regulatory miRNAs of TGFBR3. We obtained 37 miRNAs expressed in macrophage exosomes from published reports [22]. In the two sets of data (Fig. 5f), there was only one miRNA in the intersection of the two sets of data, namely miR-501-3p. Hence, we speculated that TGFBR3 was regulated by miR-501-3p.

**Table 3:** A total of 249 genes are differentially expressed in PDAC samples

Accession	Platform	Organism	Gene/miRNA	Sample
GSE91035	GPL22763	Homo sapiens	Gene	27 PDAC tissue and 8 normal pancreatic tissue
GSE71989	GPL570	Homo sapiens	Gene	14 PDAC tissue and 8 normal pancreatic tissue
GSE109110	GPL18451	Homo sapiens	Gene	The PANC-1-alone control group under normoxio (NPC groups) and the PANC-1-cocultured TAMs group under normoxio (NPM groups)

### MiR-501-3p overexpression promotes migration, invasion and tube formation of PDAC cells by down-regulating TGFBR3

**Fig. 6:**
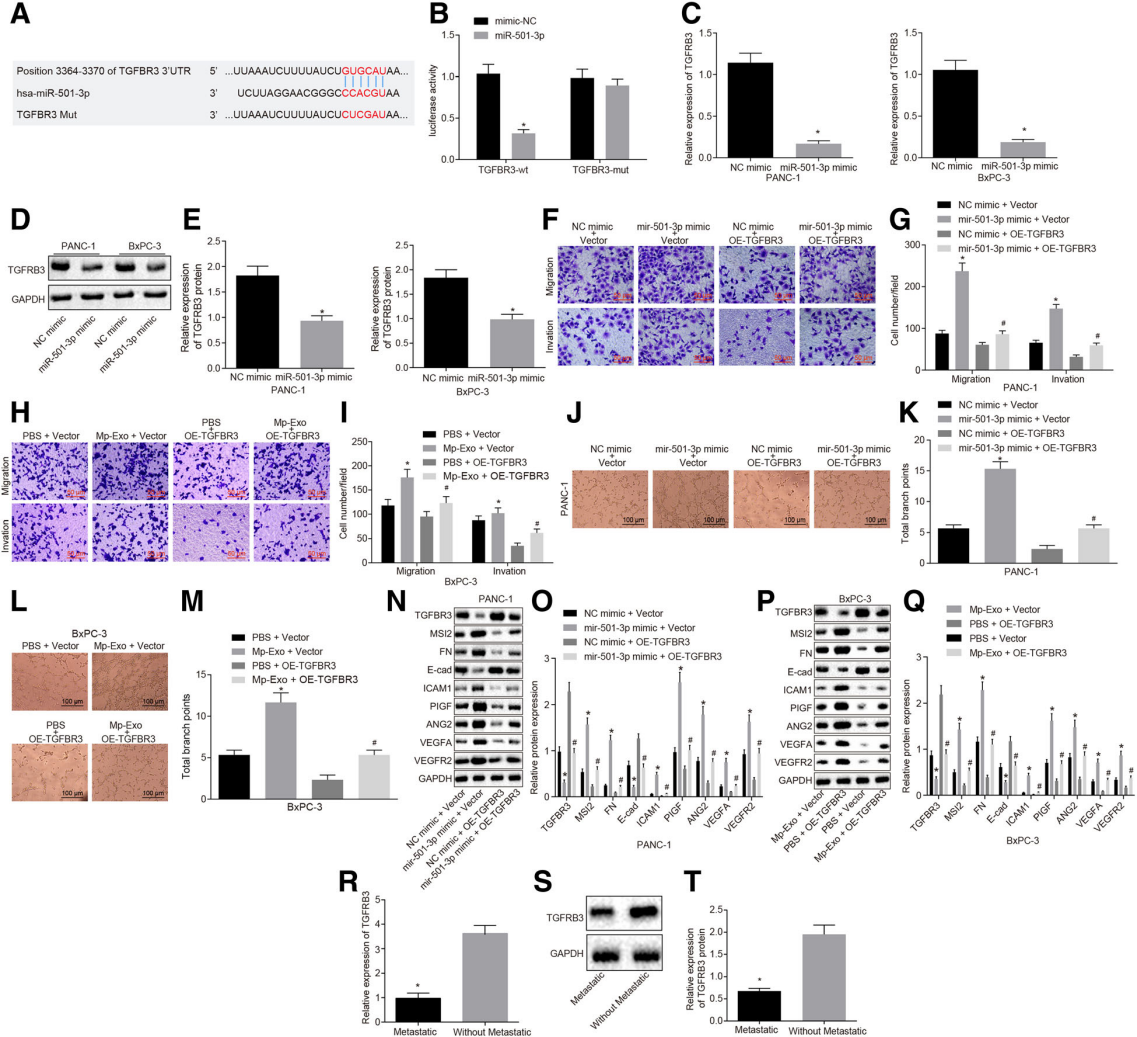
MiR-501-3p promotes migration, invasion and tube formation of PDAC cells by down-regulating TGFBR3. **a** the predicted binding sites of miR-501-3p and TGFBR3 on TargetScan database. **b** the binding of miR-501-3p to TGFBR3 was confirmed by dual luciferase reporter gene assay. * *p* < 0.05 vs. mimic-NC. C. RT-qPCR detection of TGFBR3 mRNA levels in PANC-1 and BxPC-3 cells treated with miR-501-3p mimic. * *p* < 0.05 vs. NC mimic. **d** and **e** Western blot analysis of TGFBR3 protein in PANC-1 and BxPC-3 cells treated with miR-501-3p mimic. * *p* < 0.05 vs. NC mimic. **f-i** migration and invasion abilities of PANC-1 and BxPC-3 cells treated with miR-501-3p mimic vector, miR-501-3p mimic + oe-TGFBR3, Mp-Exo + vector, PBS + oe-TGFBR3 and Mp-Exo + oe-TGFBR3 were measured by Transwell assay (200 ×). * *p* < 0.05 vs. NC mimic + vector, PBS + vector. # *p* < 0.05 vs. NC mimic + oe-TGFBR3, PBS + oe-TGFBR3. **j-m** In vitro tube formation assay was used to detect HMEC-1 cell tube formation ability and quantitative analysis in response to miR-501-3p mimic vector, miR-501-3p mimic + oe-TGFBR3, Mp-Exo + vector, PBS + oe-TGFBR3 and Mp-Exo + oe-TGFBR3 (Scale bar = 100 μM). * *p* < 0.05 vs. NC mimic + vector, PBS + vector. # *p* < 0.05 vs. NC mimic + oe-TGFBR3, PBS + oe-TGFBR3. **n-q** Western blot analysis was used to detect migration, invasion and angiogenesis-related proteins in response to miR-501-3p mimic vector, miR-501-3p mimic + oe-TGFBR3, Mp-Exo + vector, PBS + oe-TGFBR3 and Mp-Exo + oe-TGFBR3. * *p* < 0.05 vs. NC mimic + vector, # *p* < 0.05 vs. NC mimic + oe-TGFBR3, PBS + oe-TGFBR3. R. RT-qPCR detection for mRNA expression of TGFBR3 in metastatic and non-metastatic PDAC tissues (*n* = 14–42). * *p* < 0.05 vs. non-metastatic tissue. S and T. Western blot analysis was used to detect TGFBR3 protein in metastatic and non-metastatic PDAC tissues (*n* = 14–42). * *p* < 0.05 vs. tissues without metastatic. The measurement data were expressed as mean ± standard deviation. The comparison between two groups was performed by independent sample *t* test. One-way ANOVA was employed for comparison among multiple groups, followed by Tukey’s post-hoc test. Cell experiments were repeated three times

The binding sites of miR-501-3p and TGFBR3 were predicted using the TargetScan database, the 3364–3370 bases of TGFBR3–3’UTR region may be binding sites to miR-501-3p (Fig. 6a). The dual luciferase reporter gene assay confirmed that TGFBR3 was a target of miR-501-3p. The relative luciferase activity was decreased in co-transfection of pGL3-TGFBR3-Wt with miR-501-3p mimic, compared with the control of pGL3-vector (*p <* 0.05), and there was no significant difference in luciferase activity in co-transfection of pGL3-TGFBR3-Mut with miR-501-3p mimic (*p >* 0.05), indicating that miR-501-3p was bound to the TGFBR3 gene (Fig. 6b). The expression of TGFBR3 after transfection of miR-501-3p mimic was determined through RT-qPCR and Western blot analysis to further verify the targeting relationship. The results demonstrated that the mRNA (Fig. 6c) and protein levels (Fig. 6d & e) of TGFBR3 were significantly down-regulated in the PANC-1 and BxPC-3 cells in the presence of miR-501-3p mimic (*p <* 0.05). The aforementioned results consistently indicated that TGFBR3 was a target gene of miR-501-3p.

Following treatment with Mp-Exo and miR-501-3p mimic, proliferation, migration and invasion of PANC-1 and BxPC-3 cells were facilitated. In order to assess whether overexpression of TGFBR3 could inhibit the effect of Mp-Exo and miR-501-3p mimic, a series of experiments were performed. Transwell and in vitro tube formation assays revealed that overexpression of TGFBR3 can enhance the expression of TGFBR3 (Additional file 1: Figure S1B), and also reversed the effects of Mp-Exo and miR-501-3p mimic on PANC-1 and BxPC-3 cell migration, invasion and tube formation (Fig. 6f-m) (*p <* 0.05). Western blot analysis of migration, invasion and angiogenesis-related proteins revealed that there was a significant down-regulation in the expression of ICAM-1, MSI2, Fibronectin, VEGFA, VEGFR2, ANG2 and PIGF and that of E-cadherin was up-regulated in the presence of miR-501-3p mimic NC and TGFBR3 adenovirus, when compared with miR-501-3p mimic NC and TGFBR3 NC (Fig. 6n-q) (*p <* 0.05). The overexpressed TGFBR3 could reverse the effects of miR-501-3p and Mp-Exo on PANC-1 and BxPC-3 cells, as well as HMEC-1 tube formation. The expression of TGFBR3 in metastatic and non-metastatic PDAC tissues was further examined by RT-qPCR and Western blot analysis: the mRNA and protein levels of TGFBR3 were down-regulated in metastatic PDAC tissues (Fig. 6r-t) (*p <* 0.05). The aforementioned results indicate that Mp-Exo carrying miR-501-3p, by targeting TGFBR3, can promote the proliferation, migration and invasion of PANC-1 and BxPC-3 cells, in addition to the tube formation of HMEC-1 cells.

### TGF-β signaling pathway participates in the effect of miR-501-3p on PDAC cells

**Fig. 7:**
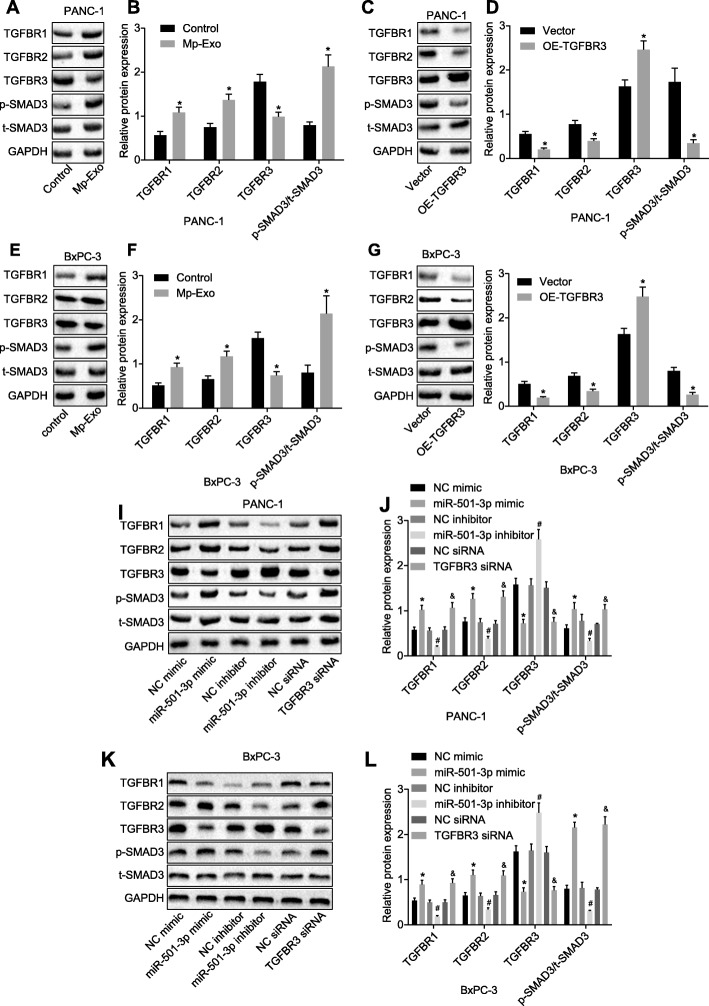
TGF-β signaling pathway is involved in the effect of miR-501-3p on PDAC cells. **a** and **b** Western blot analysis was used to detect the TGF-β signaling pathway-related proteins in PANC-1 cells after Mp-Exo treatment. **c** and **d** Western blot analysis was applied to detect the TGF-β signaling pathway-related proteins in PANC-1 cells treated with oe-TGFBR3. **e** and **f** Western blot analysis was conducted to detect the TGF-β signaling pathway-related proteins in BxPC-3 cells after Mp-Exo treatment. **g** and **h** Western blot analysis was used to detect the TGF-β signaling pathway-related proteins in BxPC-3 cells treated with oe-TGFBR3. **i** and **j** Western blot analysis was applied to detect the TGF-β signaling pathway-related proteins in PANC-1 cells after treatment of miR-501-3p mimic, inhibitor or TGFBR3 siRNA. **k** and **l** Western blot analysis was used to detect the TGF-β signaling pathway-related proteins in BxPC-3 cells after treatment of miR-501-3p mimic, inhibitor or TGFBR3 siRNA. * *p <* 0.05 vs. control, vector or NC mimic. # *p <* 0.05 vs. NC inhibitor. & *p <* 0.05 vs. NC siRNA. The measurement data were expressed as mean ± standard deviation. Data between two groups were analyzed by independent sample *t* test. One-way ANOVA was employed for comparison among multiple groups, followed by Tukey’s post-hoc test. Cell experiments were repeated three times

Studies have documented that TGFBR3 can regulate the development of various tumors through the TGF signaling pathway [17, 28]. In order to understand the molecular mechanism of miR-501-3p on PDAC, based on the pertinent reports, we examined the changes of the TGF-β signaling pathway under different treatments (Fig. 7). Mp-Exo (Fig. 7a, b, e, f), miR-501-3p mimic and TGFBR3 siRNA (Fig. 7i, j, k, l) were capable of up-regulating the levels of TGFBR1, TGFBR2 and p-SMAD3 in PANC-1 and BxPC-3 cells, and down-regulating TGFBR3 expression (*p <* 0.05). On the contrary, overexpression of TGFBR3 (Fig. 7c, d, g, h) or miR-501-3p inhibitor in PANC-1 and BxPC-3 cells up-regulated TGFBR3 expression and inhibited the levels of TGFBR1, TGFBR2 and p-SMAD3. In summary, macrophages deliver miR-501-3p through exosomes in PDAC cells, thereby targeting and regulating TGFBR3 expression and ultimately affecting the development of PDAC through the TGF-β signaling pathway. The results from in silico prediction were consistent with our experimental results.

### Inhibition of miR-501-3p in M2 macrophage-derived exosomes represses tumor formation and metastasis in vivo

**Fig. 8:**
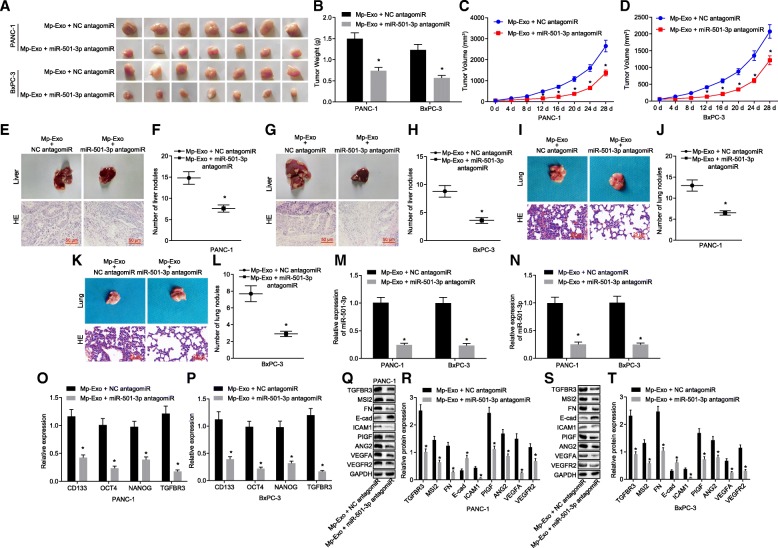
Inhibition of miR-501-3p in macrophage exosomes represses tumor formation and metastasis in nude mice. **a-d** xenograft tumors and quantitative analysis of tumor mass after Mp-Exo + miR-501-3p antagomiR treatment (*n* = 7). M2 macrophages were transfected with NC antagomiR or miR-501-3p antagomiR, after which exosomes were extracted and injected into the nude mice. Tissue samples were collected after 4 weeks to detect relevant indicators. **e-l** HE staining analysis and quantitative analysis of liver and lung nodules after Mp-Exo + miR-501-3p antagomiR treatment (n = 7). **m** and **n**. RT-qPCR detection of the expression of miR-501-3p in subcutaneous tumor tissues and exosomes from the serum of nude mice in response to Mp-Exo + miR-501-3p antagomiR. **o** and **p** RT-qPCR detection of expression of tumor cell stemness-related genes and THFBR3 in mouse subcutaneous tumor tissues in response to Mp-Exo + miR-501-3p antagomiR. **q-t** Western blot analysis of migration, invasion and angiogenesis-related proteins as well as TGFBR3 protein in PANC-1 and BxPC-3 cells treated with Mp-Exo + miR-501-3p antagomiR. * *p <* 0.05 vs. Mp-Exo + NC antagomiR. The measurement data were expressed as mean ± standard deviation. Data between two groups were analyzed by independent sample *t* test. One-way ANOVA was employed for comparison among multiple groups. The data at different time points were compared using repeated measures ANOVA, followed by Tukey’s post-hoc test

Two PDAC cell lines (PANC-1 and BxPC-3) were used to establish nude mouse models of subcutaneous and metastatic PDAC, respectively. Exosomes extracted from M2 macrophages transfected with NC antagomiR or miR-501-3p antagomiR were injected into the nude mice, and tissue samples were collected 4 weeks later. Versus those injected with M2 macrophages transfected with NC antagomiR, the weight and volume of subcutaneous tumors in the nude mice injected with M2 macrophages transfected with the miR-501-3p antagomiR were significantly decreased (Fig. 8a-d) (*p* < 0.05), and the number of liver and that of lung nodules in the nude mouse models of metastatic PDAC were significantly reduced (Fig. 8e-l) (*p* < 0.05). In addition, miR-501-3p levels were inhibited in subcutaneous tumor tissues and serum exosomes (Fig. 8m & n) (*p* < 0.01). The mRNA levels of genes (CD133, OCT4, and NANOG) related to stemness of cancer cells were significantly down-regulated (Fig. 8o & p) (*p* < 0.05). The expression of migration and angiogenesis-related proteins and TGFBR3 was determined by Western blot analysis, demonstrating significantly down-regulated expression of ICAM-1, MSI2, Fibronectin, VEGFA, VEGFR2, ANG2 and PIGF, as well as up-regulated E-cadherin and TGFBR3 in nude mice injected with M2 macrophages transfected with miR-501-3p antagomiR (Fig. 8q-t) (*p* < 0.05). The aforementioned findings indicated that the inhibition of miR-501-3p levels in M2 macrophage exosomes can suppress the tumorigenic ability of PANC-1 and BxPC-3 cells, as well as tumor metastasis ability, and also inhibit the expression of tumor cell stemness-related genes to some extent.

## Discussion

**Fig. 9:**
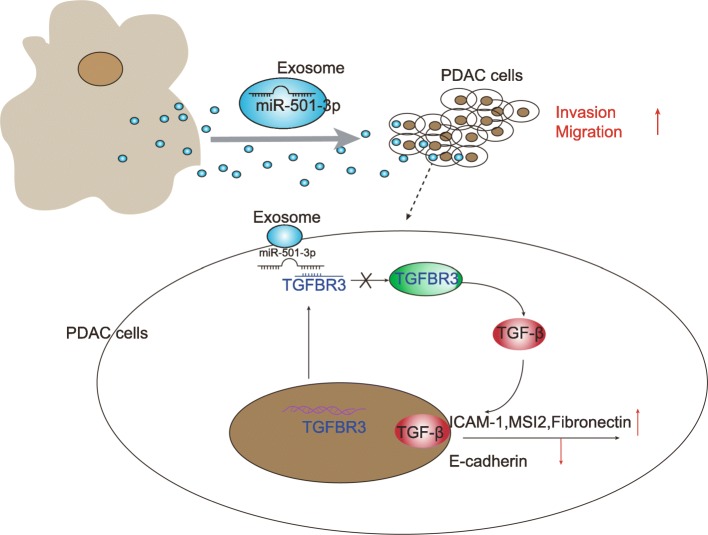
Schematic representation of macrophage-derived exosomal miR-501-3p in PDAC and the involvement of TGFBR3-mediated TGF-β signaling pathway. M2 macrophages deliver miR-501-3p through exosomes in PDAC cells, thereby down-regulating TGFBR3 expression, and ultimately accelerate the development of PDAC via the TGF-β signaling pathway, corresponding to enhanced PDAC cell invasion and migration

There is a growing number of evidences suggesting the treatment of PDAC remains a challenge, due to the susceptibility to early systemic metastasis and invasion into the adjacent vascular structures [29, 30]. Hence, it is urgent to identify minimally invasive biomarker to assist to diagnose PDAC early and develop effective treatment regimens to curtail the malignancy characterized by the high morbidity and low survival rate [13]. Tumor-derived exosomal miRNAs exert important physiological functions and are significant participants in TAM infiltration and M2 polarization [31]. In the present study, we showed that M2 macrophages deliver miR-501-3p through exosomes in PDAC cells, thereby down-regulating TGFBR3 expression, and ultimately accelerating the development of PDAC via the activation of the TGF-β signaling pathway (Fig. 9).

TAMs have dual influence on the occurrence and development of cancers according to the activation status, and the anti-tumoral and pro-tumoral effects are induced by classically activated (M1) and alternatively activated (M2) phenotypes, respectively [32]. One of the findings from our study revealed that the M2 phenotype was the main phenotype involved in TAM recruitment in PDAC, which was associated with metastasis and cumulative survival rate. M2-polarized TAMs have been highlighted as critical regulators of the correlation of cancer with inflammation. Moreover, the M2-polarized TAMs are associated with the promotion of epithelial-mesenchymal transition in pancreatic cancer [33]. In our study, we also found that Mp-Exo was shown to promote the migration and invasion of PDAC cells, as well as tumor formation and liver and lung metastasis in nude mice. The exosomes secreted from PDAC cells could stimulate the liver metastasis in nude mice by inducing pre-metastatic niche formation in the liver [34]. Moreover, a previous study provided an opposing view to the previous one suggesting that, when the PDAC cells were transfected with miR-encoding plasmid, the altered tumor-derived exosomes could control the polarization of macrophage phenotypes, making genetic therapies potential treatment options for pancreatic cancers [35].

Since highly expressed miR-501-3p was observed in PDAC tissues and M2 macrophages of PDAC cells, we performed a series of in vitro and in vivo experiments to assess the role of miR-501-3p in the function of M2 macrophage. TAM-targeted miRNA delivery has been evaluated in a preliminary study conducted by Liu et al. for anti-cancer therapy, which suggested that tumor suppression can be obtained through the repolarization of TAMs to anti-tumor M1 phenotype [36]. We also found that exosomal miR-501-3p can accelerate migration, invasion and tube formation of PDAC cells. The oncogenic role of miR-501 has also been reported in cervical cancer, where the effects of miR-501 in facilitating cell proliferation, migration and invasion, in addition to lymph node metastasis were demonstrated [16]. In addition, hepatocellular carcinoma tissues and cell lines have also been found to have up-regulated miR-501-5p, and overexpression of miR-501-5p could enhance tumor cell proliferation by targeting CYLD [37]. The study by Goto et al. defined increased expression of three miRNAs (miR-191, − 21, −451a) enclosed in serum exosomes as early diagnostic and progression markers of pancreatic cancer [38].

To further probe into the mechanism, in silico analysis was conducted to predict differentially expressed genes and regulatory miRNAs related to PDAC treated with M2 macrophages, which identified that poorly expressed TGFBR3 in PDAC and its expression could be further decreased in response to M2 macrophage treatment. Specific binding sites were confirmed between regulatory miR-501-3p and the 3’UTR of TGFBR3, which was also verified by our experimental results. MiR-501-3p overexpression could promote PDAC cell migration, invasion and tube formation by down-regulating TGFBR3. The tumor suppressive role of TGFBR3 has been proven in multiple malignancies including clear-cell renal cell carcinoma, in which the loss of TGFBR3 expression was found to be significantly involved in the tumor formation and metastasis through TGF-β-mediated mechanism [17]. The up-regulation of TGFBR3 expression significantly attenuates the motility and invasion in vitro and tumor formation in vivo of prostate cancer cells [39]. In addition, restoring the expression of TGFBR3 could result in the inhibition of invasion ability of tumor cells in vitro, as well as angiogenesis and metastasis in vivo through the inactivation of the TGF-β signaling pathway in breast cancer cells [40]. Consistently, our study revealed that the TGF-β signaling pathway was implicated in the effect of miR-501-3p-mediated downregulation of TGFBR3 on PDAC cells, which indicated a conserved anti-oncogenic role of TGFBR3 in combination with results in other cancers.

Finally, the results from the in vivo experiments in nude mice revealed that the inhibition of miR-501-3p in M2 macrophage exosomes resulted in the suppression of tumor formation and metastasis in the liver and lung. In pancreatic cancer, infiltration of M2-polarized TAM in regional lymph nodes has the capability of accelerating nodal lymphangiogenesis through the generation of vascular endothelial growth factor C and induction of regional lymph node metastasis [41]. It has been reported that miR-501-5p shares an association with poor survival of gastric cancer patients and its up-regulation facilitates cancer cell stemness by activating the Wnt/β-catenin signaling pathway [42]. In addition to miR-501-5p, corroborating evidence demonstrated that macrophages-derived exosomes can also deliver miR-21 inhibitor to gastric cancer cells and suppress the migration ability. Notably, versus conventional transfection, miRNAs delivered by exosomes are less toxic to the host cells, which is a key finding that can be a promising area of interest for future clinical trials [43].

## Conclusion

In conclusion, the aforementioned findings demonstrated that exosomes secreted by TAMs transfer miR-501-5p into PDAC cells promoted the invasion, migration and tube formation of PDAC cells through the down-regulation of TGFBR3 via the TGF-β signaling pathway activation. Therefore, exosomes from TAMs with up-regulated levels of miR-501-5p could be potential therapeutic pathway for PDAC. However, due to the insufficiency in the investigation on the role and mechanism of exosomal miR-501-5p in the angiogenesis and microenvironment of PDAC, further experiments are required to further elucidate the intrinsic mechanisms of exosomal miR-501-5p.

## Additional files

Additional file 1: **Figure S1.** Expression of miR-501-3p and TGFBR3. A. expression of miR-501-3p in pancreatic cancer cell lines after miR-501-3p mimic and miR-501-3p inhibitor treatment detected by RT-qPCR. B. the expression of TGFBR3 in pancreatic cancer cell lines after miR-501-3p mimic and miR-501-3p inhibitor treatment detected by RT-qPCR. (EPS 398 kb)

Additional file 2: **Figure S2.** Expression of M1 and M2 macrophage marker genes in exosomes. Expression of Arginase, CD206, CD68 and iNOS was detected by RT-qPCR. (EPS 348 kb)

## Data Availability

The datasets generated/analysed during the current study are available.

## Abbreviations

ANG2: Angiopoietin 2
BCA: Bicinchoninic acid
GAPDH: Glyceraldehyde-3-phosphate dehydrogenase
HE: Hematoxylin-eosin
HMEC: Human microvascular endothelial cell
HRP: Horseradish peroxidase
miR-301a-3p: microRNA-301a-3p
miR-501: microRNA 501
miR-501-3p: microRNA-501-3p
miRNAs: microRNAs
Mp-Exo: M2 macrophage-derived exosomes
MSI2: Musashi RNA binding protein 2
Mut: Mutation
NC: Negative control
OS: Overall survival
PDAC: Pancreatic ductal adenocarcinoma
RIPA: Radioimmunoprecipitation assay
RT-qPCR: Reverse transcription quantitative polymerase chain reaction
SDS-PAGE: Sodium dodecyl sulfate-polyacrylamide gel electrophoresis
SPF: Specific pathogen-free
STR: Short tandem repeat
TAM: Tumor-associated macrophage
TAMs: Tumor-associated macrophages
TGFBR3: TGF-beta Receptor III
Wt: Wild type
